# Ageing in place readiness in Thailand: urban and rural comparison

**DOI:** 10.1186/s12877-025-06864-y

**Published:** 2025-12-05

**Authors:** Faron Hattapradit, Sureeporn Punpuing, Kanchana Tangchonlatip, Piyawat Katewongsa, Yothin Sawangdee, Sutthida Chuanwan

**Affiliations:** Institute for Population and Social Research, Mahidol University Salaya, Nakhon Pathom, Putthamonthon 73170 Thailand

**Keywords:** Ageing in place, Urban, Rural, Older population

## Abstract

**Background:**

The concept of ageing in place is an important guideline for implementing support for an ageing society. This study examined the ageing index in place of residence in urban and rural areas at the national level.

**Method:**

Samples were Thai citizens age 60 years old and above by using national secondary data from the 2021 survey of the elderly population in Thailand. The Ageing in Place Index was calculated including 5 components (place, social networks, support, technology, personal characteristics).

**Results:**

The score for the Ageing in Place Index was higher in rural areas than in urban areas, at a high level (0.810) and at a moderate level (0.798) respectively. The indices for place and personal characteristics are at a moderate level, while the component for support, social networks, and technology are at a high level. The index score for social networks and technology is at a high level. The social networks index, with one sub-indicator being community participation, had a percentage score of 54.99% in rural areas, which is considered ‘*good*,’ compared to 46.70% in urban areas, which is considered ‘*fair*.’ Urban areas, however, had a technological readiness that is more suitable for ageing in place.

**Conclusions:**

Thai older in rural areas can ageing in place better than their counterparts in urban areas. Therefore, the government should promote social participation policies for the older in urban areas, and technology for the older in rural areas. Overall, Thailand needs to ensure that there is a robust support system for ageing in place so that the older enjoy an optimal quality of life.

## Introduction

The concept of Ageing In Place (AIP) is an important new idea that has been adopted as policy in several countries to address the current ageing society situation. Many countries in regions around the world have an increasing proportion older population of the total, such as North America (23.70%) and Europe (26.23%). Australia and Oceania (17.66%) and Asia (13.74%) [1]. AIP refers to promoting the idea that older individuals should remain in their original homes and communities, living independently and cohabitating with family members to care for one another for as long as possible. This concept aligns with the principles of the Madrid International Plan of Action on Ageing: MIPPA [2]. The key feature of this approach is the emphasis on shared care among families, the older, and the home community, rather than placing the burden of care solely on one group, as seen in institutional care where the government primarily provides support. Several countries have adopted the concept of AIP to develop policies that support an ageing society according to their own national contexts, such as Finland, Netherlands, UK, Japan, Singapore, and Spain [3].

The concept of AIP includes six aspects: place, community support, technology support, health care service, health care workforce, and family caregivers. The policies for supporting the ageing society in some Asian countries have begun to align with the concept of AIP, but only minimally and insufficiently given their rapidly ageing populations. That viewpoint is based on the results of a comparative study of three countries with high proportions of older populations, including Japan (35.84% of the national population age 60 + years), Singapore (22.96%), and Thailand (20.00%). It was found that Japan had the most studies on health care service (24 studies), followed by Singapore (4 studies). Thailand had the least, with one study each related to place and community support [4]. Therefore, it can be seen that the operations concerning the older that support the concept of AIP in Thailand are still very limited. As the concept of AIP is new in Thailand and has not been formally integrated into policy, there is limited data on various aspects of the older, which poses a constraint on the outcomes of AIP in Thailand. This dearth of information presents a challenge in developing programs that are in line with the concept of AIP, which is part of the Sustainable Development Goals (SDGs) such as ‘Sustainable Cities and Communities’ (No.11), ‘Poverty’ (No.1), ‘Good Health and Well-being’ (No.2), and ‘Peace, Justice, and Strong Institutions’ (No.16) [5].

Currently, Thailand has become a “complete-aged society,” (i.e., 20% or more is age 60 + years) and it is estimated that Thailand will become a “super-aged society” (20% or more is age 60 + years) in the year 2040 (with 31.5% of the population age 60 + years) [1]. Implementing the concept of AIP is extremely challenging because the older population in different areas, both urban and rural, have different lifestyles. The living conditions and important contextual factors vary significantly. In urban areas, for instance, the structure is complex, with advanced and modern infrastructure and technology that facilitate daily life. However, the population density is high, and the urban lifestyle is fast-paced, focusing on work and driving the local economy, leading to competition for job security. By contrast, rural areas have a lower population density, and the residents generally have closer-knit relationships than their urban counterparts [6], making it easier to access and manage the development of the quality of life for the older population. This could be a highlight, as the concept of AIP emphasizes the importance of staying in the home and community where individuals grew up, fostering a bond with the place and people [7]. Additionally, individuals have a significant positive relationship with their environment [8].

The governance structure of Thailand is divided into central and regional systems. At the provincial level, there are two types of local administrative organizations (LAO) managing the development of the population’s quality of life: (1) Municipalities, which are further divided into three levels: city municipalities (population of 50,000 or more), town municipalities (population of 10,000 or more), and sub-district municipalities (population of 7,000 or more). These municipalities must be sufficiently developed and have adequate local revenue collection; and (2) Sub-district Administrative Organizations (SAO). This SAO is the smallest type, and mostly is found in a rural context, with less development and a low volume of local revenue collection [9]. The two areas are different, with the municipal districts having a clear level of urbanization. Therefore, it can be seen that these differences will affect the implementation of ageing policies in their respective areas, especially in translating policy into practice. The readiness and capabilities vary, including personnel, resources, budgets, operational policies of leaders, and local management. This results in disparities in the care provided by the community, particularly for vulnerable groups such as the older who require age-appropriate care. Assessing the readiness for AIP according to the context of urban and rural areas is one approach to planning for the implementation of AIP operations, using accurate and reliable databases that align with the local context. This study examined the AIP Index (AIPI) in urban and rural areas among a Thai older population.

## Methods

### Data source

The data for this study were collected using a cross-sectional survey of the Thai older population: The Aging in Place Index Project used secondary data from the 2021 round of the Survey of the Older Population in Thailand (SOPT), This is a database from the National Statistical Office (NSO) of Thailand that surveys data related to the older every 3 years. This is the 6th time. Data were collected during October to December, 2021. This research limited the study population to respondents age 60 years old and above (Mean = 69.59, S.D.=7.88), resulting in a sample size of 43,777 people (209,409 people from SOPT) [10].

### Data management and calculation AIPI

The data was divided into two contextual areas: (1) Urban, i.e., those living within a municipal district; and (2) Rural, i.e., those living outside a municipal district. The AIPI in this study consists of five components: (1) *PlaceIndex (PI)* 4 sub-indicators; 1)Households with Bedrooms Suitable to the Older (HBSO); 2) Households with Bathrooms Suitable to the Older (HBASO); 3)Households with Grab Bars in the Bathroom (HGBB); 4)House Stability (HS); *(2)Social Networks Index (SNI)* 4 sub-indicators; 1)Participation in Community Activities (PCA); 2)Cohabitation with Family; Relatives, and/or Others (CFRO); 3)Receiving Visits from Offspring (RVO); 4)Financial Support Received from Offspring (FSRO); *(3)Support Index (SI)* 4 sub-indicators; 1) Receiving Medical Welfare (RMW); 2)Access to Home and Community Health Services (AHCHS); 3)Receiving Care from Formal Caregivers (RCFC); 4)Receiving Informal Care (RIC); *(4)Technology Index (TI)* 5 sub-indicators; 1)Telephone Communication Usage (TCU); 2)Social Media Usage (SMU); 3)Home Appliances (HA); 4)Reading-Writing Competency (RWC); 5)Receiving Useful Information (RUI) *(5)Personal Characteristics Index (PCI)* 4 sub-indicators; 1)Older Individuals Without Dependency (OIWD); 2)Happiness in Life (HL); 3)Sufficiency of Income (SI); 4)Working Individuals Age 60–64 (WIA 60–64). For this study, the AIPI was adapted from the concept of Active and Independent Participation (AIP) proposed by Pani-Harreman [11], who conducted a systematic scoping review on the components and meaning of AIP, through a properly and reliably developed process by Hattapradit [12]. Moreover, the concept developed by Pani-Harreman was adopted because it aligns well with the Thai social context, and its indicators are considered both reliable and standardized, thus ensuring conceptual consistency and measurement validity in this study. The composite index analysis used the Aggregate Z score Method [13]. The sum of the index values for each component was calculated using the average of the indicators.

$$\mathrm{Index}={\textstyle\sum_{\mathrm i=1}^{\mathrm n}}\frac{{\overline{\mathrm x}}_{\mathrm i}}{{\mathrm M}_{\mathrm i\ast\mathrm n}}$$

When ${\overline X}_i=$ Average score of indicators I, $\:{\mathrm{M}}_{\mathrm{i}}$= The maximum value of the indicator answers at I, **n** = The number of indicators in each component. Following the analysis of the index’s 5 components (PI, SNI, SI, TI, PCI), based on the aforementioned formula, we proceeded to analyze the overall AIPI as follows:

$$\mathrm{Ageing}\;\mathrm{In}\;\mathrm{Place}\;\mathrm{Index}\;(\mathrm{AIPI})=\frac{\mathrm{PI}+\mathrm{SNI}+\mathrm{SI}+\mathrm{TI}+\mathrm{PCI}}5$$

### Interpretation

The results were interpreted using a proportional scoring method based on four levels by Faramnuayphol [14], who studied the index (Active aging assessment tool for the Thai older) in the past, related to the quality of life of the older. whereby: (1) Score percentage 00.00–25.00 indicates ‘low’ level (MI = 1); (2) Score percentage 25.01–50.00.01.00 indicates ‘moderate’ level (MI = 2); (3) Score percentage 50.01–75.00.01.00 indicates ‘good’ level (MI = 3); and (4) Score percentage 75.01–100.00 indicates ‘very good’ level (MI = 4). The AIPI score has a potential value ranging from 0 to 1, meaning that, if the index value approaches 1, it indicates that the older have a ‘high’ capability for AIP, while if the index value approaches 0, it indicates that the older have a ‘low’ capability for AIP. In sum, the AIPI score is categorized into three levels: (1) Low (0.000–0.499.000.499); (2) Moderate (0.500–0.799.500.799); and (3) High (0.800–1.000.800.000) [15].

## Results

The study found that the AIPI in rural and urban areas across five dimensions, specifically the social networks index, showed a difference in one sub-indicator: PCA was higher for the older in rural areas than those in urban areas. The values for the other four dimensions (PI, SI, TI, PCI) were not significantly different at the statistical-measurement level, but there was a trend in the percentage of index values that differed (Table 1). When considering each sub-indicator of the AIPI components, the results of the analysis can be summarized as follows:

1. *Place:* The values for sub-indicators that show the readiness of important home locations between the two areas found that households with bathrooms suitable for the older and grab bars in the bathroom was higher in urban than in rural areas. Meanwhile, households with bedrooms suitable for the older had a lower percentage score in urban than in rural areas.
2. *Social networks: *The sub-indicator regarding the readiness of social networks PAC in rural areas had an index measure of 54.99 (MI = 3), which is higher than in urban areas, or 46.70 (MI = 2). Other sub-indicators also show higher percentage scores in rural compared to urban areas.
3. *Support:* The values for all four sub-indicators regarding readiness for support were higher in rural than in urban areas.
4. *Technology:* For technological readiness, all five sub-indicators had higher percentage scores in urban than in rural areas. This result highlights the advantage of technological advancement in facilitating AIP.
5. *Personal characteristics:* The values for the sub-indicators that reflect readiness indicate that life satisfaction and income sufficiency scores were higher in urban than in rural areas. However, the employment of individuals age 60–64 years had higher percentage scores in rural than in urban areas.

**Table 1:** Ageing in place index by urban and rural area

Indicator	Urban			Rural		
	*N*	PS	MI	*N*	PS	MI
1. Place						
• HBSO	18,315 (23,126)	79.20	4	17,613 (20,300)	86.76	4
• HBASO	16,133 (23,126)	69.76	3	11,239 (20,300)	55.36	3
• HGBB	2,209 (14,759)	14.49	1	1,457 (12,762)	11.42	1
• HS	14,735 (14,759)	99.84	4	12,729 (12,771)	99.67	4

2. Social Networks						
• PCA	9,420 (20,170)	46.70	2	9,592 (17,442)	54.99	3
• CFRO	16,960 (20,170)	84.09	4	15,142 (17,442)	86.81	4
• RVO	10,319 (15,330)	67.31	3	9,734 (14,276)	68.18	3
• FSRO	5,699 (7,021)	81.17	4	5,583 (6,747)	82.75	4

3. Support						
• RMW	18,886 (19,010)	99.35	4	16,168 (16,240)	99.56	4
• AHCHS	10,377 (18,878)	54.97	3	10,197 (16,151)	63.14	3
• RCFC	11,250 (19,010)	59.18	3	11,131 (16,240)	68.54	3
• RIC	1,575 (1,735)	90.78	4	1,426 (1,555)	91.70	4

4. Technology						
• TCU	9,097 (13,181)	69.02	3	7,805 (12,200)	63.98	3
• SMU	5,797 (13,181)	43.98	2	4,188 (12,200)	34.33	2
• HA	11,440 (17,063)	67.05	3	9,197 (14,748)	62.36	3
• RWC	15,255 (17,063)	89.40	4	12,565 (14,748)	85.20	4
• RUI	15,414 (16,573)	93.01	4	13,303 (14,359)	92.65	4

**5. Personal characteristics**						
• OIWD	23,342 (23,652)	98.69	4	20,435 (20,692)	98.76	4
• HL	6,983 (18,194)	38.38	2	5,484 (16,083)	34.10	2
• SI	14,736 (23,738)	62.08	3	11,808 (20,763)	56.87	3
• WIA 60–64	4,492 (7,820)	57.44	3	4,457 (67.50)	67.50	3

Note: Households with Bedrooms Suitable to the Older (*HBSO*), Households with Bathrooms Suitable to the Older (*HBASO*), Households with Grab Bars in the Bathroom (*HGBB*), House Stability (*HS*)

Note: Participation in Community Activities (*PCA*), Cohabitation with Family; Relatives, and/or Others (*CFRO*), Receiving Visits from Offspring (*RVO*), Financial Support Received from Offspring (*FSRO*)

Note: Receiving Medical Welfare (*RMW*), Access to Home and Community Health Services (*AHCHS*), Receiving Care from Formal Caregivers (*RCFC*), Receiving Informal Care (*RIC*)

Note: Telephone Communication Usage (*TCU*), Social Media Usage (*SMU*), Home Appliances (*HA*), Reading-Writing Competency (*RWC*), Receiving Useful Information (*RUI*)

Note: Older Individuals Without Dependency (*OIWD*), Happiness in Life (*HL*), Sufficiency of Income (*SI*),Working Individuals Age 60–64 (*WIA 60–64*)

When analyzing the scores of the AIPI related to home and environment components of the index (PI, SNI, SI, TI), it was found that the AIPI in rural areas is higher than in urban areas, with both indices being at a ‘high’ level, 0.825 and 0.809, respectively. Additionally, when analyzing the AIPI scores across all five components, the AIPI in rural areas is also higher than in urban areas, with values of 0.810 at a ‘high’ level and 0.798 at a moderate level, respectively (Table 2).

**Table 2:** Overview of the ageing in place index of the older population by urban and rural area

Components of Index	Urban		Rural	
	Index	Results	Index	Results
1. Place Index; PI	0.750	Moderate	0.750	Moderate
2. Social Networks Index; SNI	0.813	High	0.875	High
3. Support Index; SI	0.875	High	0.875	High
4. Technology Index; TI	0.800	High	0.800	High
5. Personal Characteristics Index; PCI	0.750	Moderate	0.750	Moderate
Total of component Index 1–4	0.809	High	0.825	High
Total of component Index 1–5	0.798	Moderate	0.810	High

Note: Components 1–4 consider the readiness for AIP from an environmental perspective, Component Index 1–5 considers the readiness for AIP based on environmental and personal characteristics

## Discussion

The analysis of the AIPI of the older population, with comparison by urban and rural area of residence, shows the readiness of the older to AIP. In particular, the social network component differs at the index measurement level. As for other components, although there is no difference at the index measurement level, there are trends in percentage scores in different domains as follows:

### Urban areas

The older living in these areas are preparing for AIP, which is both significant and challenging, as it is a component with limitations.

#### Social networks

 For this dimensions, the AIPI is lower than in rural areas due to the differences in the urban context, which is complex and has specific urban living patterns. Most people focus primarily on work and income. Although there are many residents, the relationships are not as close as in rural areas. This results in fewer opportunities for the older to participate in community activities (PCA), leading to weaker bonds with others in the community. The relationship with the community depends on the context of the place and the duration of residence, reflecting the value placed on community relationships by the older [6]. Additionally, the differences in individual factors among the older affect their PCA, which serve as a basis for learning [16]. A strong social network is beneficial for social participation and learning among the older.

#### Technology

For this indicator, living in an urban area stands out as an advantage. All five sub-indicators (TCU, SMU, HA, RWC, RUI) show a higher percentage score in urban compared to rural areas. Areas with urban characteristics are more technologically equipped to facilitate learning for the older. Naturally, the skills in using these technologies have been accumulated through daily life experiences within the urban social context in the past. This enables the older to use technology conveniently and constructively in their daily life, with a higher usage rate than in rural areas. Technological advancements have brought satisfaction to the older, and the city’s development has comprehensively impacted the provision of various services [17]. However, complex technology can be a barrier to learning, causing older individuals to lose interest. Additionally, age-related decline in sensory perception (e.g., hearing, vision, reading) poses obstacles to using technology [18]. This is particularly true for middle-aged seniors (70–79 years) and older seniors (80 + years), who use technology less frequently [19]. One of the impacts to be wary of is the potential for crime related to technology, as older individuals living in urban areas are more likely to become targets of cyber-criminals.

#### Place

For this domain, the analysis found that there is readiness of home facilities to accommodate the older. The indicators of HBSO, HGBB have higher percentage scores in urban than in rural areas. This indicates that urbanization facilitates better readiness in terms of facilities. For example, newer homes are built with structural stability and designed to accommodate the older inhabitants, especially with toilets that are sit-down types and higher than the floor in urban areas at 69.76% compared to 55.36% in rural areas. This difference perhaps reflects the implementation of policies supporting an ageing society that is increasingly living in cities. In Thailand, urban areas have specific policies to design and construct housing that is age-friendly [20]. In addition, public places in urban areas are increasingly being designed for the older in mind, with a coverage of 75.00%, which is higher than the national average of 70.81% [21]. One important factor is the economic status of older families. Most have sufficient financial resources to build a proper residence according to age-friendly principles. That said, modifying homes to suit the older usually incurs extra expenses, which is a limitation, especially for resource-constrained households [22]. On the other hand, older homes in urban areas benefit from urban planning, with distances from homes being relatively closer to convenience stores, restaurants, pharmacies, public spaces, public transportation for travel, and places for business [23]. The distance to hospitals and clinics [17] is also conducive for AIP.

#### Personal characteristics

 The values of the sub-indicators for this dimension of readiness show that happiness in life and sufficient income were higher in urban than in rural areas. This finding suggests that the advancement of urban characteristics can provide older individuals with the opportunity to generate income sufficient to cover their expenses. Economic status is a crucial factor affecting the well-being of older individuals living in urban areas, which, of course, have higher costs than rural areas [24]. This is consistent with a previous study which identified factors associated with Thai older leaving the full-time workforce including higher education level, urban residence, and support rates [25].These factors indicate that older individuals living in an urban area contribute to the development of personal factors, which include higher education, stable career opportunities, financial preparedness, and satisfaction in various aspects of life, leading to greater happiness and overall improved quality of life. It is evident that many of the features of urban areas enable older individuals to attain a higher AIP in their home community.

### Rural areas

The readiness for AIP has significant strengths and challenges.

#### Social network

This dimension is clearly significant, especially in terms of PCA, which is more common in rural than in urban areas. The three sub-indicators (CFRO, RVO, FSRO) have higher percentage scores for rural than urban areas, although there is no difference at the index measurement level. This indicates that Thai social networks in rural areas are strong and provide good support for the older. The social context in rural areas still relies on mutual support, and joint social activities foster trust in relationships among community members more than in large cities [6]. Data on AIP in rural China show that social support is positively related to increased life goals through social trust. Both working-age individuals and the older perceive mutual support, and social trust positively affects the mental health of the older [26]. Therefore, the fact that the social network index in rural areas is higher than in urban areas is conducive to AIP.

#### Support

 In this study, the scores of all sub-indicators (RMW, AHCHS, RCFC, RIC) in rural areas were higher than urban areas because rural areas have stronger family and community support for the older. Additionally, the government’s management makes it easier to provide healthcare services, especially with a focus on home care involving community health volunteers or other types of caregivers as the government has policies to support senior citizens. The Ministry of Public Health has developed strong primary healthcare services in vulnerable communities, using a multidisciplinary team, in accordance with the Primary Health Care Act [27]. Additionally, there is a network of public sub-district health promotion hospitals which are more accessible in rural than urban areas, other things being equal. These nearby hospitals are close to and trusted by the community, thereby facilitating access to the homes of the older, along with promoting family members to look after frail household members. This is a positive development, as the government emphasizes promoting community care within the social and cultural context of the community [28].

#### Technology

At the time of this research, this dimension remained a challenge in rural areas due to their lower score of study sub-indicators compared to urban areas. This deficiency is attributed to the low income and other personal factors that thwart adopting new technologies, along with the cultural context where rural areas tend to use technology less than urban communities [18]. Specifically, the use of social media is perceived as too complicated by the older, with younger older individuals being more likely to use social media than oldest individuals. This aligns with data showing that the percentage of older individuals in non-municipal areas who own a mobile phone, use a smart phone, and access the internet (66.9%, 80.1%, and 44.9%, respectively) is lower than in municipal areas (79.4%, 85.8%, and 62.5%, respectively). Younger older individuals had the highest usage rates of these technologies, while the middle-aged and oldest individuals had significantly lower usage rates [19]. The positive effects of using social media include enhancing social interactions among the older [29]. Technology is beneficial in connecting with society, supporting one’s daily routine, providing convenience, and offering entertainment [30].

#### Place

The older in rural areas have the advantage of bedrooms that are more age-friendly, with a higher scores compared to their urban counterparts. This is because most older people’s homes in rural areas are single-story houses, accounting for 65.08% [21]. On the other hand, older people living in dwellings that are two stories or higher prefer their bedrooms to be on the ground floor rather than an upper floor, which aligns with studies showing that the older prefer their bedrooms on the ground floor [31]. A ground-floor bedroom provides convenience for daily activities and reduces strain on the legs and joints from climbing and descending a flight of stairs. By contrast, in urban areas, space for home construction is limited, and houses or apartment buildings are often at least two stories high. In particular, Bangkok has many tall buildings that require climbing stairs, especially buildings with 1–4 stories where the law does not mandate elevators. This increases the need for daily, arduous physical movement, which poses a risk of falls for the older. Therefore, in terms of place, rural areas are more likely to have houses with bedrooms on the ground floor, making them more suitable for older residents.

#### Personal characteristics

The work-life situation of the older is more conducive for AIP in urban areas. This is because many older people in rural areas still need to work to earn enough income to cover living expenses. This supposition aligns with the results of the Thai national employment survey from 2021 to 2023, which found an increase in the proportion of older workers (35.9%, 36.1%, 37.5%), especially in areas outside municipal zones where the proportion of workers was higher than in municipal areas (41.4% and 32.2%, respectively). Generally, the proportion of older male workers is higher than their female counterparts (53.7% and 32.2%, respectively) [32]. Important factors that keep Thai older people working include gender, age, marital status, and household debt [25]. In particular, household debt is one factor that drives older people in rural areas to keep working past retirement age (e.g., 60 years) in order to make ends meet. By contrast, older people in urban areas who, upon reaching retirement age, prefer to leave daily employment, especially if they have sufficient savings to cover living expenses. These dynamics result in the phenomenon that rural areas have a higher proportion of older people in the workforce, which positively impacts the employment rate and promotes PCA. Although working in older life might be an indicator of financial constraints, it has a positive effect on generating a sense of accomplishment by earning income and enjoying the beneficial effect of participating with coworkers.

## Conclusions

The readiness for AIP between the two areas found that the AIPI was higher in rural areas than in urban areas, at a high level (0.810) and at a moderate level (0.798). Overall, of each component the AIPI in urban and rural areas are generally at a similar level. That said, there are differences in the values of the social-network sub-indicators, specifically in PCA. The percentage score differences in these sub-indicators reflect that urban communities excel in technology, place, and personal characteristics, while rural areas are distinguished by their social networks and support systems.

## Recommendations policy

The following are policy recommendations for supporting AIP: *In urban areas,* the recommendation focuses on social networks. The government should promote PCA, emphasizing the involvement of the older with others in their residential community context. *In rural areas,* the recommendation focuses on technology. The government should establish policies to support the readiness of older homes, mandating essential home assistance technology, especially those that ease physical strain for the older, thereby benefiting daily life activity. Additionally, promoting the use of smart phones and social media to facilitate daily living is advised. *For both urban and rural areas,* promoting AIP should focus on facilities. Policies should prioritize improving age-friendly bathrooms and mandating sit-down toilets and grab bars in bathrooms as a first step, covering all older households in Thailand. Regarding support, policies should emphasize home and community health services and informal care. For personal characteristics, the government should establish measures or policies to ensure the older have sufficient income and financial stability for ageing, and create integrated policies across all dimensions to enhance long-term quality of life, assessed by the happiness of daily living in the later stages of life.

## Data Availability

The datasets analyzed during the current study are available in the National Statistical Office (NSO) of Thailand repository, https://www.dop.go.th/download/knowledge/th1687612748-2406_0.pdf, reference number DS. 1504/97.
